# Incidental Solitary Fibrous Tumour of the Pleura on Trauma CT: A Radio-Pathological Correlation

**DOI:** 10.7759/cureus.97245

**Published:** 2025-11-19

**Authors:** Sai Ram Kumar Yatiraju, James A Whittaker, Akanksha Soni, Paul Bishop, Mohamed Al-Aloul

**Affiliations:** 1 Respiratory Medicine, Stockport NHS Foundation Trust, Stockport, GBR; 2 Radiology, Stockport NHS Foundation Trust, Stockport, GBR; 3 General Medicine, Stockport NHS Foundation Trust, Stockport, GBR; 4 Pathology, Manchester University NHS Foundation Trust, Manchester, GBR; 5 Pulmonary Medicine, Stockport NHS Foundation Trust, Stockport, GBR

**Keywords:** benign vs malignant, pleural neoplasms, solitary fibrous pleural tumour, stat 6 immunohistochemistry, thoracic radiology

## Abstract

Solitary fibrous tumours of the pleura (SFTPs) are rare growths that are usually benign, but their behaviour can be unpredictable. We describe the incidental discovery of an SFTP in a 39-year-old man who presented to the Emergency Department after a fall. Although he had no respiratory symptoms, trauma imaging unexpectedly revealed a pleural-based mass in the right upper chest. Further CT scans showed a 5.5 × 3 cm ovoid lesion with mixed enhancement along the right major fissure, and FDG-PET demonstrated low metabolic activity with no signs of spread. A CT-guided biopsy revealed spindle cells within a collagen-rich background and the typical “staghorn” vessels, with CD34 and STAT6 positivity confirming the diagnosis. The mass was removed via video-assisted thoracoscopic surgery, and histology confirmed a benign tumour without mitotic activity or necrosis. The patient recovered well and remained disease-free at follow-up. This case highlights how SFTPs can present unexpectedly in young, asymptomatic individuals and reinforces the importance of careful assessment of incidental thoracic findings. Although most SFTPs are curable with surgery, continued follow-up is recommended due to the occasional unpredictable behaviour of these tumours.

## Introduction

Solitary fibrous tumours (SFTs) are a rare type of mesenchymal neoplasm that can develop in virtually any anatomic site but most often arise within the thoracic cavity, with an equal distribution between men and women [1].

Most SFTs are benign; however, 10-20% display malignant features, which may lead to local recurrence or metastasis, sometimes occurring several years after diagnosis [2]. Therefore, long-term follow-up is essential. Cross-sectional tomography (CT) is the preferred initial imaging modality. On contrast-enhanced CT, SFTs exhibit notable vascularity, with approximately 65% of cases demonstrating avid contrast enhancement. Enhancement is most conspicuous in the arterial and early portal venous phases, with a contrast washout in the delayed phase.

Here, we present the case of a young man who was incidentally found to have a small right pleural-based mass on trauma CT imaging. We then detail dedicated thoracic imaging features and biopsy findings, in combination, confirming the diagnosis of a solitary fibrous tumour of the pleural (SFTP), before describing intraoperative and ex vivo tumour morphology and histology. This case is noteworthy due to the uncommon incidental detection of a benign pleural SFT in a young adult. It also highlights the importance of correlating imaging features with histopathological findings when evaluating pleural masses, reinforcing the pivotal role of multidisciplinary assessment in achieving an accurate diagnosis.

## Case presentation

**Figure 1:**
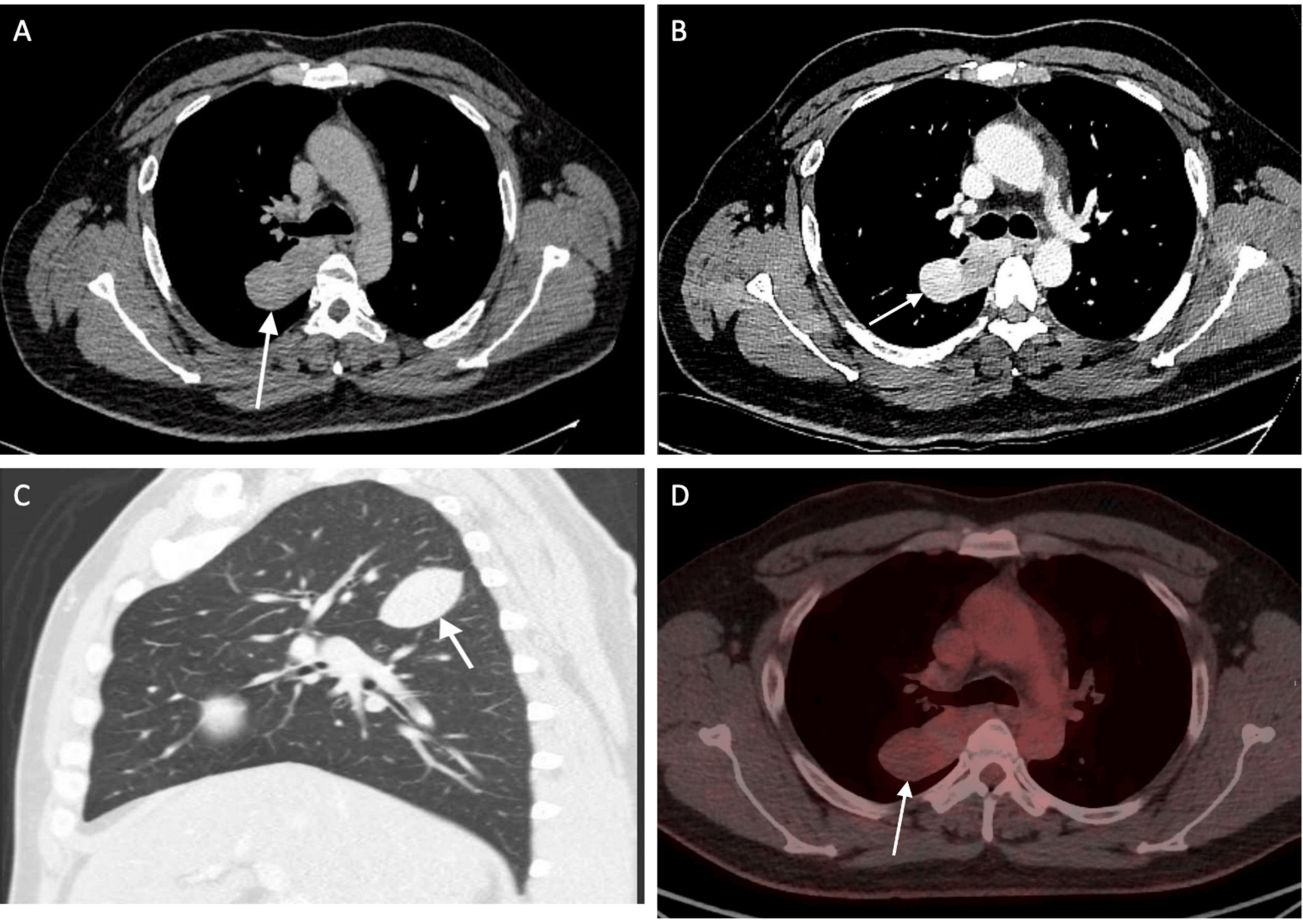
Radiological findings of the Solitary Fibrous Tumour of the Pleura A. Axial section of CT Thorax (non-contrast) showing a bilobed mass. B. Axial section of CT Thorax with contrast showing heterogeneous enhancement. C. Sagittal section of CT (lung window) showing a well-defined mass arising from the right major fissure. D. FDG PET axial window showing right upper lobe mass with low diffuse FDG avidity. FDG PET: Fluorodeoxyglucose positron emission tomography

A 39-year-old male support worker with type 2 diabetes, hypertension, and alcohol excess presented to the Emergency Department (ED) after an alcohol-related fall. A trauma assessment CT head and neck (non-contrast) incidentally showed a pleural-based lesion in the upper right hemithorax (Figure 1A). This finding prompted referral to the chest clinic for an urgent suspected lung cancer pathway for further evaluation.

At the chest clinic, the patient was asymptomatic, with no respiratory or systemic symptoms, including fever, night sweats, or weight loss. He could walk long distances and climb stairs without limitation (WHO Performance Status 0). Social history included a seven-pack-year smoking history (quit two weeks prior) and daily alcohol use.

On examination, he was alert and afebrile, with normal vital signs and a body mass index (BMI) of 25 kg/m². There was no clubbing or palpable extra thoracic lymphadenopathy. Cardiovascular and respiratory examinations were unremarkable. A subsequent dedicated thoracic CT, performed on a dual-energy scanner, at 60 seconds (portal venous phase), demonstrated a 5.5 × 3 cm ovoid, well-defined pleural-based mass along the superior aspect of the right major fissure, with heterogeneous internal enhancement, lying close to major mediastinal vessels, and no pleural effusion (Figure 1B, Figure 1C). 

On fluorodeoxyglucose positron emission tomography (FDG PET), the mass showed diffusely low avidity (SUV max 2.3; mediastinal blood pool 2.4) without abnormal uptake elsewhere (Figure 1D).

Given the radiological appearance, a CT-guided biopsy was performed, revealing spindle cells arranged in irregular fascicles within a fibrous stroma, with thin-walled “staghorn” vessels. Immuno-histochemistry showed strong positivity for CD34 and STAT6, and negativity for AE1/AE3, SMA, and S100, confirming the diagnosis of an SFTP. 

**Figure 2:**
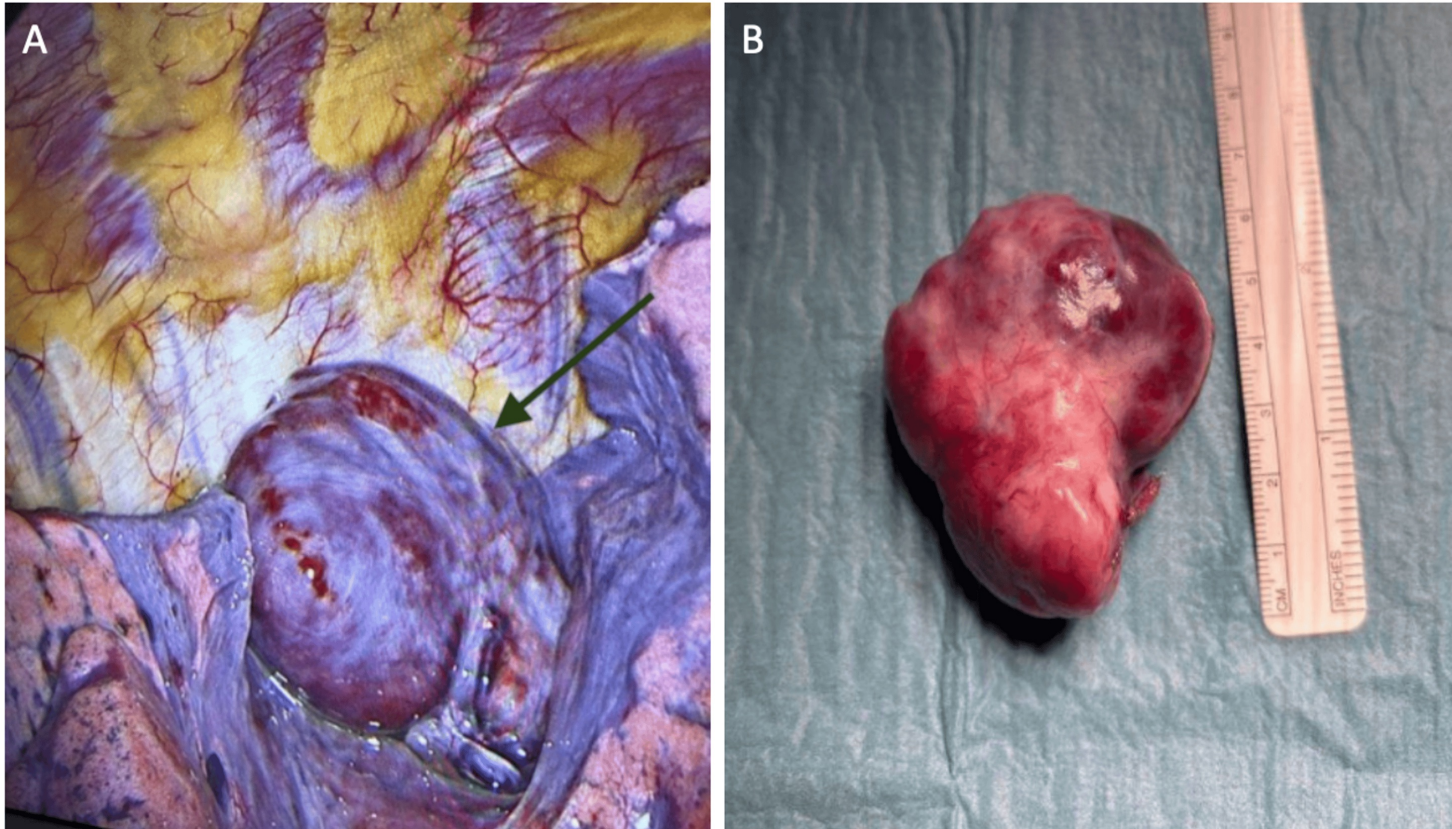
Gross Pathology Appearance of the Solitary Fibrous Tumour of the Pleura A. In vivo appearance of the solitary fibrous tumour during video-assisted thoracoscopic surgery. B. Completely resected solitary fibrous tumour of the pleura.

Following confirmation of the SFTP, a multidisciplinary review was conducted, and the patient was scheduled for definitive surgical excision via video-assisted thoracoscopic surgery. In vivo, the pleural tumour was seen arising from the apex of segment 6 on a vascular pedicle, without invading the lung parenchyma (Figure 2A). The mass was enucleated and submitted to pathology without lung parenchyma attached. Macroscopically, the tumour was a firm, congested, well-circumscribed, irregular nodule measuring 53 x 42 x 26 mm (Figure 2B). The pleural surface appeared intact. The cut surfaces in the histology laboratory appeared firm, pale, congested and cystic. Cysts measured 5 x 4 mm. 

**Figure 3:**
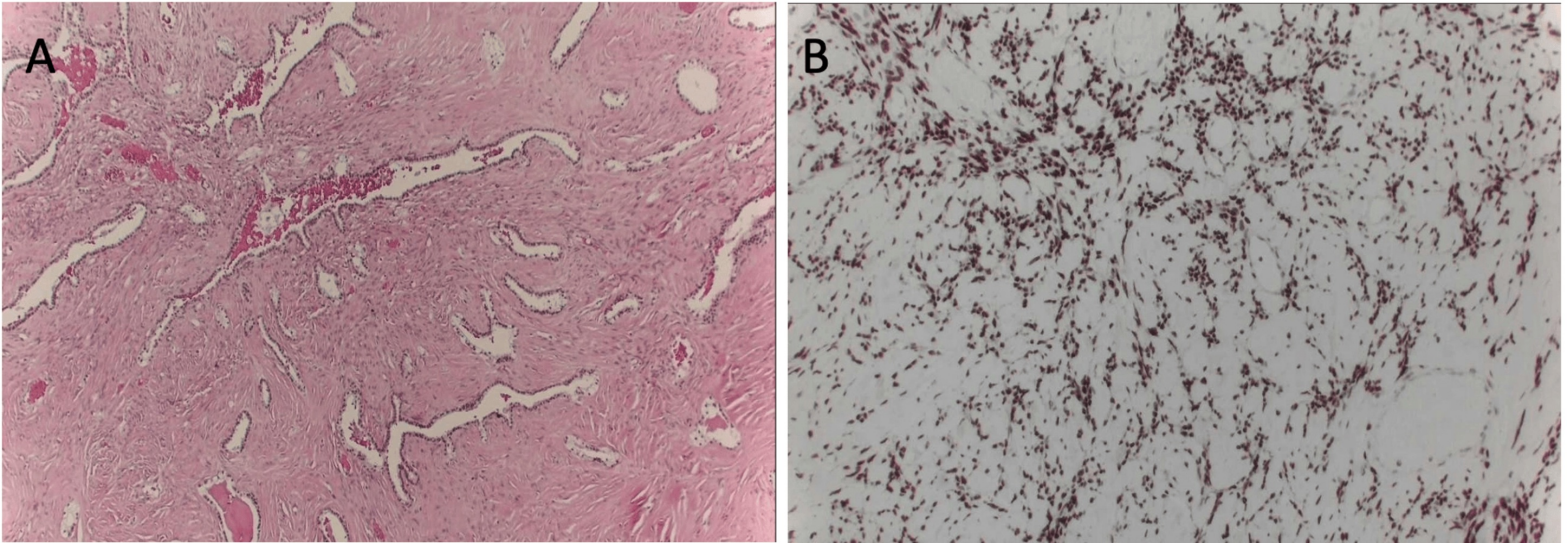
Histopathological Appearance of the Solitary Fibrous Tumour of the Pleura A. Microscopic appearance of the SFTP on H&E stain showing collagenous stroma and spindle cell proliferation. B. Microscopic appearance of the SFTP showing strong nuclear immunoreactivity for STAT6.

Microscopic examination of the excised mass revealed a spindle cell tumour of variably low to moderate cellularity. The stroma was fibrotic. Scattered within the tumour, there were clusters of glands, some of which were cystically dilated (Figure 3A). There were no mitotic activity and no necrosis. The stroma was positive for CD34, whereas CD31 stained only vessels. The epithelial element was positive for AE1/AE3 and TTF-1. The spindle cells were positive for STAT6 confirming the diagnosis of an SFTP (Figure 3B).

## Discussion

SFTPs account for less than 5% of pleural tumours and originate from submesothelial fibroblasts, not mesothelial cells [3-5]. Most SFTPs are benign, slow-growing and asymptomatic, often found incidentally on imaging, as in our patient after a trauma CT. However, about 10-20% show malignant potential, with local invasion, recurrence after surgical resection or metastasis [4]. General features of benign vs malignant SFTPs are contrasted in Table 1. Furthermore, benign lesions usually arise from the visceral pleura and are pedunculated, growing outward into the pleural cavity. Malignant tumours more often start from the parietal or diaphragmatic pleura and tend to invade the lung parenchyma [5].

**Table 1:** Features of Benign vs Malignant Solitary Fibrous Tumours of the Pleura HPF: High-Power Field; SFTP: Solitary Fibrous Tumour of the Pleura References: [1-4]

Feature	Benign SFTP	Malignant SFTP
Incidence	~80–90% of SFTPs	~10–20% of SFTPs
Growth pattern	Slow-growing, well-circumscribed	Rapid growth, infiltrative margins
Tumour size	Typically <10 cm	Often >10 cm (“giant” SFTs)
Histopathology	Low cellularity, minimal atypia, low mitotic index (<4 mitoses/10 HPF)	High cellularity, nuclear atypia, necrosis, ≥4 mitoses/10 HPF
Imaging features (CT/MRI)	Well-defined margins, homogeneous or mild heterogeneous enhancement	Lobulated or irregular margins, heterogeneous enhancement with necrotic or hemorrhagic areas
Recurrence/Metastasis	Rare after complete resection	Higher recurrence and metastatic potential (up to 60% in some series)
Prognosis	Excellent, near 100% 5-year survival	Variable; 5-year survival ~60–80% depending on completeness of resection

Radiologically, SFTPs typically appear as a well-circumscribed pleural-based mass with smooth margins and an obtuse angle to the pleural surface [3,4]. On contrast-enhanced CT, small SFTPs enhance homogeneously, while larger ones show heterogeneous enhancement [5]. In our case, thoracic CT showed a 5.5 × 3 cm pleural-based ovoid mass with heterogeneous enhancement. Although the heterogeneous enhancement raised concern for malignancy, histopathology confirmed its benign nature. Imaging features that suggest malignancy include a large tumour size, ipsilateral pleural effusion, parietal pleural attachment, and loss of tumour pedunculation [3,5]. Tumour size remains one of the most reproducible prognostic markers, with lesions exceeding 10 cm, so-called giant SFTs, more often exhibiting malignant histological features [4]. Nevertheless, large size does not always correlate with an aggressive clinical course and radiological features alone cannot reliably distinguish benign from malignant lesions [5]. PET is not routinely used. When it is, high FDG uptake may indicate malignancy [3]. In our patient, the small tumour size, pedunculation, low FDG uptake and absence of pleural effusion supported a benign lesion.

Histologically, SFTPs show a storiform pattern of spindle cells in a collagenous stroma with branching staghorn vessels. Immunohistochemistry showing diffuse nuclear CD34 and STAT6 positivity confirms the diagnosis, as in our case. This finding distinguishes SFTPs from other spindle-cell pleural tumours [4,5]. Histological features which portend a higher risk of recurrence or metastasis such as hypercellularity, high mitotic activity, necrosis and nuclear pleomorphism were all absent in the excised tumour from our patient.

The mainstay of treatment is complete surgical excision with negative margins, which is both diagnostic and the choice of treatment in most benign cases [3,4]. Complete excision results in a low risk of local recurrence and metastasis. Long-term recurrence rates vary, less than 2% for benign non-pedunculated tumours, up to 14% for benign pedunculated forms, and between 14% and 63% for malignant lesions [5]. Even after complete resection, recurrences have been reported, reinforcing the need for ongoing radiological surveillance. Current evidence supports follow-up with serial chest imaging every 6-12 months initially and annually thereafter [1]. In our patient, given the lesion's small size, radiologically well-defined margins and benign histopathology, video-assisted thoracoscopic surgical excision was considered the optimal approach. Post-operative monitoring will focus on excluding recurrence on follow-up imaging.

## Conclusions

Although most SFTPs are benign, their radiological variability and malignant potential require thorough evaluation. Our case, found incidentally on trauma CT in an asymptomatic young male, illustrates the diagnostic challenge and underscores the need for histopathological confirmation to guide management.

Complete surgical excision remains the treatment of choice in most SFTs. However, given their potential for late recurrence or malignant transformation, long-term radiological surveillance is essential. Multidisciplinary collaboration between radiologists, pulmonologists, thoracic surgeons and pathologists ensures accurate diagnosis and optimal patient outcomes.
